# Non-suicidal self-injury and emotional burden among university students during the COVID-19 pandemic: cross-sectional online survey

**DOI:** 10.1192/bjo.2022.616

**Published:** 2022-12-01

**Authors:** Daniel Schleicher, Elisa Heidingsfelder, Stephanie Kandsperger, Irina Jarvers, Angelika Ecker, Romuald Brunner

**Affiliations:** Clinic of Child and Adolescent Psychiatry, Psychosomatics and Psychotherapy, University of Regensburg, Germany

**Keywords:** COVID-19, non-suicidal self-injury, self-harm, university students, childhood and adolescence

## Abstract

Owing to restrictions of the COVID-19 pandemic, increased stress is evident in university students with a lifetime history of non-suicidal self-injury (NSSI). Therefore, we examined two groups of university students (*n* = 174) in an online survey, one that exhibited early NSSI in adolescence (*n* = 51) and another that exhibited continuous NSSI beyond the age of 18 (*n* = 123) (German Clinical Trials Register DRKS00023731). We compared the two groups in terms of depressive symptoms in the previous 2 weeks as well as self-perceived changes in emotional burden, urge to self-injure and NSSI frequency in the first year of the pandemic compared with the year before (pre-pandemic). Among other findings, both groups showed an increase in emotional burden and urge to self-injure.

Non-suicidal self-injury (NSSI) refers to the intentional infliction of injury to one's own body, but without the intent to die as a result.^1^ In contrast to suicidal self-injury, the main function of NSSI seems to be affect regulation.^2^ Prevalence rates range from 7.5% to 46.5% in adolescence, from 4% to 23% in adulthood and 38.9% among university students.^2^ Previous studies show that early-onset and repetitive NSSI in childhood and adolescence is related to higher levels of stress, further self-injury and emotion regulation problems in young adulthood.^3,4^ Young adults and especially university students with a lifetime history of self-injury are at increased risk for the development of depression and severe distress during the COVID-19 pandemic lockdown.^5,6^ To our knowledge, there have been no studies comparing the psychological impact due to the pandemic between university students who self-injured only during childhood and adolescence (eNSSI: early NSSI, before 18 years of age) and those who continued to self-injure during early adulthood (cNSSI: continuous NSSI, before and after 18 years of age). Therefore, the purpose of this study and preliminary report is to examine depressive symptoms and self-perceived changes in the emotional burden, urge to self-injure and NSSI frequency during the pandemic within and between both groups. Data were collected as part of a larger online survey, which was conducted from April to August 2021. The main research question of the larger online survey relates to characteristics of NSSI, previous risk as well as protective factors and their associations with depression, aggressiveness and emotional reactivity in current young adulthood. This study is pre-registered in the German Clinical Trials Register (DRKS00023731), where further information on methodology and psychometrics can be found. Because of the online survey's collection period (approximately 1 year after the onset of the pandemic) and the aim of investigating various risk factors, such as (chronic) stressful events, we included these additional questions to assess self-reported changes in the experience regarding NSSI during the current pandemic in this at-risk student population.

## Method

The survey was conducted using the PsyToolkit platform.^7,8^ After introducing inclusion criteria and study objectives, digital informed consent to participate was obtained from all participants. Inclusion criteria were: university student status, age (18–25 years), sufficient understanding of the German language, and lifetime history of NSSI. There were no exclusion criteria other than not meeting the inclusion criteria. Demographic data and general information on NSSI (e.g. frequency, duration) were collected. Perceived changes in the three variables (emotional burden, urge to self-injure and NSSI frequency) during the pandemic compared with the year before were measured using self-constructed items (Table 1). In addition, for comparison of depressive symptoms over the previous 2 weeks, the German version of the Brief Patient Health Questionnaire (PHQ-9) was used.^9^ Trigger warnings and contact information for counselling centres were regularly displayed. Participation was voluntary and could be discontinued at any time. The authors assert that all procedures contributing to this work comply with the ethical standards of the relevant national and institutional committees on human experimentation and with the Helsinki Declaration of 1975, as revised in 2008. All procedures involving human participants/patients were approved by the Ethics Committee of the University of Regensburg (reference number: 20-2041-101).

**Table 1:** Descriptive statistics (upper section) and results of between-group Mann-Whitney *U*-tests and within-group one-sample Wilcoxon signed rank tests for the COVID-19 items and PHQ-9 score (lower section)^a^

Variable	Question	Response category	Group, *n* (%)		Total, *n* (%)
			eNSSI	cNSSI	
NSSI frequency 1 (year with most frequent NSSI during childhood and adolescence)	‘Please try to remember the year you most often self-injured as a child/teenager. How often did you do this in that year?’	1 time	3 (5.90)	3 (2.40)	6 (3.40)
		2 times	3 (5.90)	4 (3.30)	7 (4.00)
		3 to 5 times	18 (35.30)	20 (16.30)	38 (21.80)
		More than 5 times	27 (52.90)	96 (78.00)	123 (70.70)
NSSI frequency 2 (last year, during the pandemic)	‘Last year, during the COVID-19 pandemic, how often did you intentionally injure yourself?’	Not at all	−	36 (28.60)	36 (28.60)
		1 time	−	13 (10.30)	13 (10.30)
		2 times	−	19 (15.10)	19 (15.10)
		3 to 5 times	−	18 (14.30)	18 (14.30)
		More than 5 times	−	37 (29.40)	37 (29.40)
Last NSSI episode	‘When was the last time you did this (NSSI)?’	Last week	−	21 (17.10)	21 (12.10)
		Last month	−	27 (22.00)	27 (15.50)
		Last year	−	41 (33.30)	41 (23.60)
		2 to 3 years ago	12 (23.50)	27 (22.00)	39 (22.40)
		4 to 5 years ago	18 (35.30)	5 (4.10)	23 (13.20)
		6 years ago or longer	21 (41.20)	2 (1.60)	23 (13.20)
Medical treatment	‘Has this behaviour ever resulted in hospital admission or injury so severe that medical treatment was required?’	No	51 (100.00)	105 (85.40)	156 (89.70)
		Yes	−	18 (14.60)	18 (10.30)
Psychiatric treatment	‘Have you ever received psychiatric, psychotherapeutic or psychological treatment for this behaviour?’	No	39 (76.50)	74 (60.20)	113 (64.90)
		Yes	12 (23.50)	49 (39.80)	61 (35.10)
Suicidality	‘Was the main reason for your self-injury the intention to die by suicide?’	Never	47 (92.20)	105 (85.40)	152 (87.40)
		Occasionally	4 (7.80)	18 (14.60)	22 (12.60)
		Always	−	−	−
Suicide attempt	‘Have you attempted suicide in your life?’	No	49 (96.10)	91 (74.00)	140 (80.50)
		Yes, before the age of 18	2 (3.90)	18 (14.60)	20 (11.50)
		Yes, after the age of 18	−	4 (3.30)	4 (2.30)
		Yes, before and after the age of 18	−	10 (8.10)	10 (5.70)

Dependent variable	Question	Response category	Group	*n*	Mean	s.d.	Mean rank	Test statistic	*P*	Effect size
Self-reported change in emotional burden	‘Last year, during the COVID-19 pandemic, did you perceive a higher emotional burden compared with the year before?’	Significantly lower (−2); Somewhat lower (−1); Equal/not applicable (0); Somewhat higher (+1); Significantly higher (+2)	Total	174	1.03	0.93	eNSSI = 86.00, cNSSI = 88.12	*U* = 3060.00	0.788	*η^2^* = 0.00
			eNSSI	51	1.00	0.92		*t* = 952.50	<0.001	*r* = 0.72
			cNSSI	123	1.04	0.94		*t* = 4413.00	<0.001	*r* = 0.71
Self-reported change in urge to self-injure	‘Last year, during the COVID-19 pandemic, had the urge to self-injure increased compared with the year before?’	Significantly decreased (−2); Somewhat decreased (−1); Equal/not applicable (0); Somewhat increased (+1); Significantly increased (+2)	Total	174	0.43	0.89	eNSSI = 76.24, cNSSI = 92.17	*U* = 2562.00	0.037	*η^2^* = 0.02
			eNSSI	51	0.25	0.69		*t* = 100.50	0.016	*r* = 0.34
			cNSSI	123	0.50	0.95		*t* = 1895.50	<0.001	*r* = 0.44
Self-reported change in NSSI frequency^b^	‘Last year, during the COVID-19 pandemic, did you injure yourself more often compared with the year before?’	Significantly rarer (−2); Somewhat rarer (−1); Equal/not applicable (0); Somewhat more often (+1); Significantly more often (+2)	cNSSI	123	0.30	0.91		*t* = 1199.00	<0.001	*r* = 0.31
PHQ-9 score	Questionnaire to assess depressive symptoms over previous 2 weeks	Not at all (0); Several days (1); More than half the days (2); Nearly every day (3)	Total	159	12.67	6.68	eNSSI = 63.22, cNSSI = 87.26	*U* = 1858.50	0.002	*η^2^* = 0.06
			eNSSI	48	10.25	6.45				
			cNSSI	111	13.72	6.54				

NSSI, non-suicidal self-injury; eNSSI, early non-suicidal self-injury (before 18 years of age); cNSSI, continuous non-suicidal self-injury (before and after 18 years of age); PHQ-9, Brief Patient Health Questionnaire.

a. Hypothesis tests were two-sided; α = 0.05. The possible and present range of response categories for all COVID-19 items (Self-reported change in emotional burden, Urge to self-injure and NSSI frequency) was 4 (from −2 to +2). The present range for PHQ-9 score was 26 (from 0 to 26; maximal possible range from 0 to 27).

b. Questions about (self-reported change in) NSSI frequency during the pandemic were not applicable to the eNSSI group and therefore not reported.

In total, *n* = 240 people started the online survey; *n* = 174 of these could be included in the final analysis (eNSSI group: *n* = 51, 46 female, mean age 21.53 years (s.d. = 1.80, range 19–25); cNSSI group: *n* = 123, 104 female and 1 diverse, mean age 21.91 years (s.d. = 1.97, range = 18–25)). As regards age, there was no significant difference between the two groups (*U* = 2773.00, *P* = 0.225). In total, *n* = 66 people had to be excluded: did not meet inclusion criteria (*n* = 3), contradictory responses (*n* = 1), questionnaire not started or discontinued (*n* = 48), and NSSI only in adulthood (*n* = 14). University students from different fields of study (economics, *n* = 12; legal studies, *n* = 8; mechanical and electrical engineering, *n* = 4; medical studies, *n* = 16; natural sciences, *n* = 26; psychology, *n* = 14; social sciences, *n* = 18; teaching, *n* = 32; other and not specified, *n* = 44) and study programs (Bachelor, *n* = 98; Master, *n* = 24; state exam, *n* = 52) participated.

As data were not normally distributed and the two groups were of different sizes, group differences were analysed using non-parametric Mann-Whitney *U*-tests. Changes in the studied variables during the pandemic within both groups were calculated using one-sample Wilcoxon signed rank tests.

## Results

The cNSSI group showed a significantly higher depression score and reported a significantly greater urge to self-injure, whereas there was no difference in perceived change of emotional burden between the two groups. Furthermore, there was a significant increase within both groups in perceived urge to self-injure and emotional burden during the pandemic compared with the previous year. The cNSSI group also reported a significant increase in NSSI frequency. Descriptive statistics and an overview of the presented results can be found in Table 1.

## Discussion

Continuation of NSSI beyond adolescence appears to be a risk factor for increased depressive symptoms and urge to self-injure as well as more frequent self-injury during the pandemic. Nonetheless, even with early cessation of self-injury in adolescence, a self-reported increase in emotional burden and urge to self-injure is evident during the pandemic. On the one hand, the results of our survey support prior findings that university students with a history of self-injurious behaviours in general are at high risk for experiencing distress during the COVID-19 pandemic lockdown.^6^ On the other hand, our results extend previous knowledge, as the group of university students who had self-injured only in childhood and adolescence also show a specific risk due to the amplification of perceived emotional burden and urge to self-injure. Despite cessation of self-injurious behaviour in the past, these students also represent a vulnerable group to potentially resume self-injurious behaviour.

Limitations of the study are a lack of generalisability to samples other than university students and the use of subjective ratings about self-perceived changes via online survey. Moreover, it should be emphasised that this study is not a longitudinal study, but an online survey in a cross-sectional design. For future research, conducting longitudinal studies would be important to examine causal relationships. In addition, other variables should be collected in the future to characterise the sample (e.g. ethnicity, refugee status and academic performance), which would allow analyses for subgroup differences.^2,10,11^ Strengths are the anonymity of participation and using an online survey to reach a wide range of affected university students studying various subjects during the COVID-19 pandemic.

Future studies should investigate relevant protective and risk factors for NSSI in university students during the pandemic. Results to date indicate that the reduction of social stress and more time for self-care resulting from the pandemic were particularly beneficial, whereas being alone, financial distress, severity of COVID-19 infection and experiencing increased stress are seen as major risk factors.^12,13^ With many restrictions and the loss of resources due to the pandemic (e.g. social distancing and isolation, elimination of leisure activities and online learning at home), it is important that university students at increased risk for self-injury be offered various prevention and intervention options through universities and colleges.^14^ These measures could include, for example, online support for students and their families as well as campaigns to strengthen the sense of community within the university.^14^ The use of screening procedures for important risk factors (e.g. financial problems, urge to self-injure, problematic alcohol use, experience of abuse, and depressive symptoms) by professional and trained health workers at universities would also be useful.^14–16^

## Data Availability

The data that support the findings of this study are available from the corresponding author, D.S., on reasonable request.
